# Effects of Selective African Spitting Cobra Venoms and Their Three-Finger Toxins on the Modulation of Platelet Function and Blood Clotting

**DOI:** 10.3390/toxins18090368

**Published:** 2026-08-26

**Authors:** Mahtab Khatibi, José R. Almeida, Ashifa Al Juwaiser, Soheil Gilabadi, Ketan Patel, Sakthivel Vaiyapuri

**Affiliations:** 1 School of Pharmacy, University of Reading, Reading RG6 6UB, UK; m.khatibi@pgr.reading.ac.uk (M.K.); jose.dealmeida@butantan.gov.br (J.R.A.); a.aljuwaiser@pgr.reading.ac.uk (A.A.J.); s.gilabadi@pgr.reading.ac.uk (S.G.); 2 Butantan Institute, Sao Paulo 05503-900, Brazil; 3 School of Biological Sciences, University of Reading, Reading RG6 6UB, UK; ketan.patel@reading.ac.uk

**Keywords:** African spitting cobras, *Naja mossambica*, *Naja nigricincta*, *Naja pallida*, three-finger toxins, cytotoxins, haemostasis, platelets, blood coagulation

## Abstract

Venoms of African spitting cobras, including *Naja mossambica*, *N. nigricincta*, and *N. pallida*, are widely recognised for their cytotoxicity. However, their effects on platelets and blood coagulation remain poorly characterised. To address this gap, we characterised their enzymatic profiles and evaluated their effects on haemostasis and cell viability using functional blood-based and cellular assays, respectively. All three venoms markedly impaired haemostatic regulation by affecting intrinsic coagulation, significantly inhibiting platelet function, and inducing haemolysis. They also exerted potent myotoxic effects on cultured myoblasts/myotubes while causing only minimal cytotoxicity towards platelets. Fractionation by reversed-phase HPLC followed by mass spectrometry identified three-finger toxins that reproduced the potent platelet-inhibitory and myotoxic activities of the crude venoms but only partially accounted for the haemolytic and anticoagulant effects. These findings demonstrate that African spitting cobra venoms induce coordinated, multifaceted effects on human blood, with three-finger toxins serving as key functional mediators alongside other potential venom components. Overall, this study advances our understanding of the effects of spitting cobra envenoming, provides a foundation for developing improved snakebite therapies, and highlights venom-derived components as potential scaffolds for developing novel antithrombotic agents.

## 1. Introduction

Snakebite envenoming remains a major yet neglected global health issue, causing an estimated 140,000 deaths annually [1]. Sub-Saharan Africa is among the regions most heavily affected, owing to its high diversity of medically important venomous snakes and limited access to appropriate treatment and effective antivenom [2,3]. Among medically important African snakes, spitting cobras of the genus *Naja* are widely distributed across the continent, with species such as *Naja mossambica*, *Naja nigricincta* and *Naja pallida* found across eastern, southern, and parts of central Africa [4,5,6]. Epidemiological studies suggest that spitting cobras account for the vast majority of reported elapid snakebite cases in these regions [7,8]. This likely reflects their frequent presence in agricultural and residential settings, increasing the likelihood of human–snake encounters [9]. As a result, bites from these species contribute substantially to hospitalisation, morbidity and mortality associated with snakebites [8,10,11]. Envenoming by African spitting cobras can produce both local and systemic clinical manifestations [10]. Locally, these venoms cause intense pain, oedema, blistering and progressive necrosis, which can lead to permanent disfigurement or long-term loss of limb function [10,12,13]. When ocular tissues are exposed to venom, keratitis may develop, and in severe cases, permanent visual impairment can occur [13]. In contrast to several non-spitting Asian cobras, whose venoms are predominantly neurotoxic and frequently cause neuroparalysis [14], envenoming by African spitting cobras is primarily characterised by cytotoxic responses [8,15]. Systemic manifestations also include cardiovascular impairment and disturbances of haemostasis [16,17,18].

These clinical features are consistent with venom proteomic patterns showing a higher abundance of cytotoxic three-finger toxins (3FTxs) [19]. Proteomic profiles of *N. mossambica*, *N. nigricincta* and *N. pallida* have identified 3FTxs as the most prevalent toxin family, with phospholipase A_2_ (PLA_2_) enzymes present at lower levels [4,20]. Cytotoxic 3FTxs interact directly with cell membranes and can disrupt membrane integrity, thereby impairing cellular and tissue integrity, including on platelets [21,22,23]. In addition, several cobra venom 3FTxs have been reported to inhibit platelet aggregation, impair primary haemostasis, and potentially elevate haemorrhagic risk [24,25]. PLA_2_ may further perturb haemostasis by hydrolysing membrane phospholipids in platelets and erythrocytes, contributing to platelet dysfunction, haemolysis, and the release of pro-inflammatory lipid mediators [26,27,28]. Collectively, these toxins can disrupt haemostasis through multiple mechanisms, including platelet lysis or inhibition, fibrinogen degradation, and modulation of clot formation and stability [24,29]. The primary treatment for spitting cobra envenomation is the administration of regionally produced polyvalent antivenom [30,31,32]. Although antivenom remains an essential life-saving intervention, it has well-documented limitations, including narrow species specificity, restricted accessibility, and limited efficacy against venom-induced local tissue damage and haemostatic disturbances [33]. Therefore, a deeper understanding of the composition and biological activities of these venoms is needed to improve antivenom efficacy and to identify better therapeutic strategies for snakebite envenoming. Accordingly, this study aimed to characterise the venoms of *N. mossambica*, *N. nigricincta*, and *N. pallida* in detail, investigate their effects on blood, platelets, and coagulation pathways, and identify and purify the specific venom components underlying their haemotoxic effects.

## 2. Results

### 2.1. Spitting Cobra Venoms Possess MP and PLA_2_ Activities

**Figure 1:**
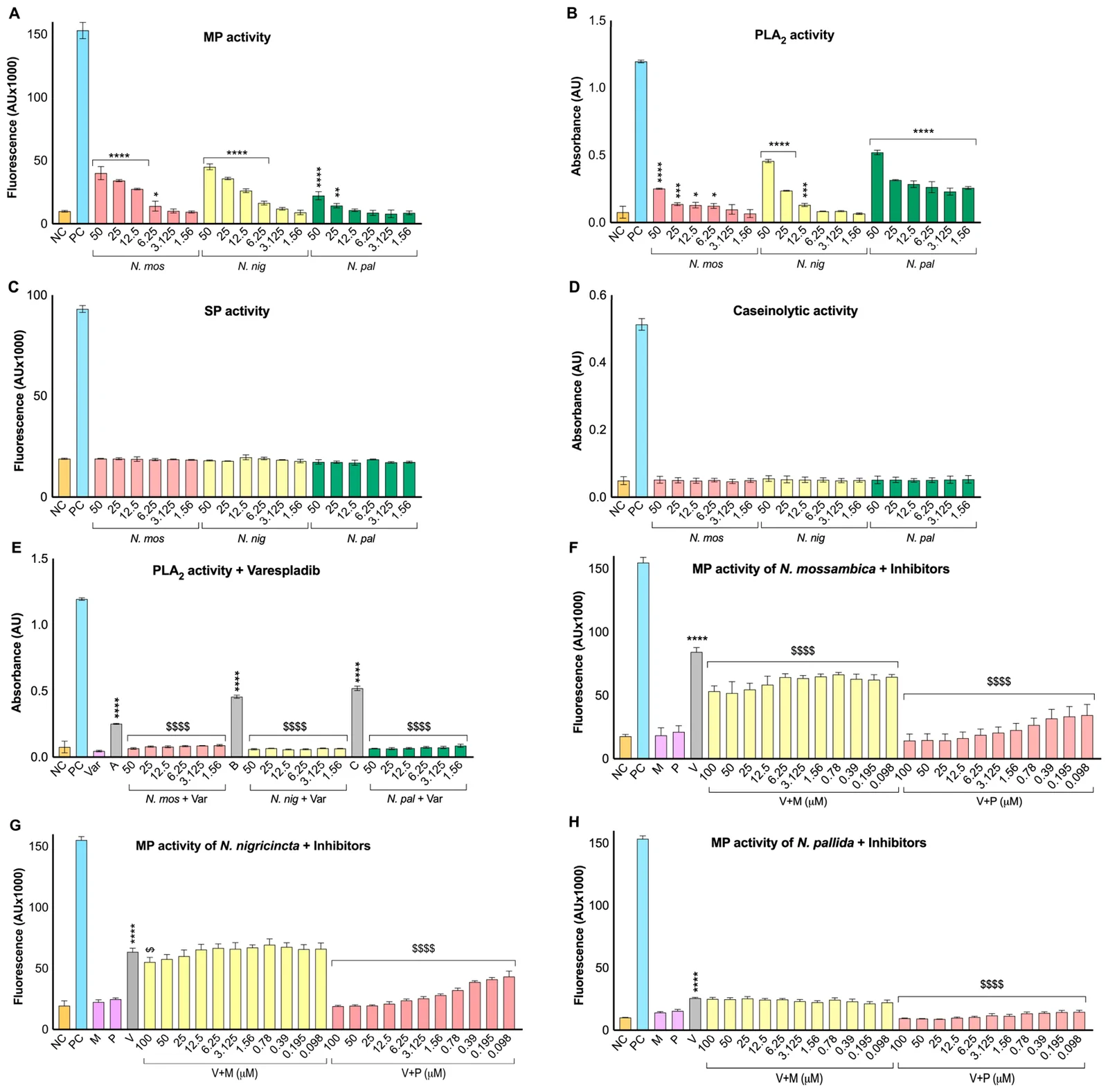
Enzymatic activities of spitting cobra venoms and the effects of specific inhibitors. (**A**) MP, (**B**) PLA_2_, (**C**) SP, and (**D**) caseinolytic activities at different concentrations of all three spitting cobra venoms were measured using appropriate substrates for each enzyme by spectrofluorimetry. Venoms are indicated as *N. mos* (*Naja mossambica*), *N. nig* (*Naja nigricincta*), and *N. pal* (*Naja pallida*). Similarly, (**E**) PLA_2_ activity of all venoms in the presence of various concentrations of varespladib (Var) was measured. Venom-alone controls included: A—*Naja mossambica*; B—*Naja nigricincta*; C—*Naja pallida*. (**F**–**H**) MP activity of *N. mossambica*, *N. nigricincta*, and *N. pallida* was measured following treatment with various concentrations of marimastat (M) or prinomastat (P). Phosphate-buffered saline (PBS) with the substrate was used as the negative control (NC) for all assays. *Crotalus atrox* venom (50 µg/mL) served as the positive control (PC) for MP, SP, and caseinolytic assays. Bee venom was used as the PC for PLA_2_ assays. (*) indicates a significant difference compared to the NC, and ($) indicates a significant difference between venom alone (V) and inhibitor-treated samples. Data are presented as mean ± S.D. (*n* = 6 individual experiments) and were analysed using one-way ANOVA with Tukey’s multiple comparisons test (* *p* < 0.05, ** *p* < 0.01, *** *p* < 0.001; and **** *p* < 0.0001) ($$$$ *p* < 0.0001).

To characterise the enzymatic profiles of various concentrations (50–1.56 μg/mL) of *N. mossambica*, *N. nigricincta*, and *N. pallida* venoms, metalloprotease (MP), serine protease (SP), caseinolytic, and PLA_2_ assays were performed using DQ gelatin, BAAMC, azocasein, and a chromogenic PLA_2_ substrate (4-nitro-3-(octanoyloxy) benzoic acid; NOB), respectively. The MP assay showed concentration-dependent activity across all three spitting cobra venoms (Figure 1A). In *N. mossambica* and *N. nigricincta*, activity at the highest concentration (50 μg/mL) was almost fourfold above the negative control (NC), followed by a gradual reduction at intermediate concentrations (25–12.5 μg/mL) and a further decrease at 6.25–3.125 μg/mL. In *N. pallida*, the highest concentration (50 μg/mL) was approximately twofold higher than NC, whereas lower concentrations (12.5–1.56 μg/mL) showed no significant MP activity. The MP activities observed in these venoms were largely lower than those in a viper (*C. atrox*) venom that was used as a positive control (PC).

Similarly, the PLA_2_ assay showed significant activity in all tested cobra venoms (Figure 1B). In *N. mossambica*, the highest concentration (50 μg/mL) induced activity that was nearly 20% of the PC (bee venom). This was followed by a gradual decrease with dilution (25–6.25 μg/mL), approaching the NC level at the lowest concentrations (3.125–1.56 μg/mL). *N. nigricincta* exerted a strong response at a concentration of 50 μg/mL, achieving around 40% of the PC activity, with a stepwise decrease (25–12.5 μg/mL). *N. pallida* also exhibited significant PLA_2_ activity across the tested range (50–1.56 μg/mL), reaching almost 50% of the PC activity at 50 μg/mL and approaching around 30–35% at the lower concentrations (25–1.56 μg/mL). In contrast, neither the SP nor the caseinolytic assay detected measurable activity at any concentration tested (50–1.56 μg/mL) in these venoms (Figure 1C,D). Overall, these findings reveal prominent MP and PLA_2_ activities in these spitting cobra venoms, with no detectable SP or caseinolytic activity across the tested concentration range.

### 2.2. Varespladib and Prinomastat Effectively Inhibit PLA_2_ and MP Activities, Respectively

To evaluate the inhibitory effects of small-molecule inhibitors on spitting cobra venom enzymes, varespladib (50–1.56 μM) was tested against PLA_2_, and marimastat and prinomastat (100–0.098 μM) were evaluated against MP in each venom at 50 μg/mL. Varespladib produced near-complete inhibition of PLA_2_ activity in all three venoms, suppressing activity to baseline across the entire concentration range relative to venom-only controls (Figure 1E). In *N. mossambica*, marimastat reduced MP activity by approximately 30% relative to the venom-only control (Figure 1F). In *N. nigricincta*, only the higher marimastat concentrations (100–50 μM) induced limited inhibition, whereas lower concentrations (25–0.098 μM) had no significant effect (Figure 1G). In *N. pallida*, marimastat showed no significant inhibition, with MP activity remaining comparable to that of the venom-alone control (Figure 1H). In contrast, prinomastat consistently produced potent, concentration-dependent inhibition across all three spitting cobra venoms. In *N. mossambica* and *N. nigricincta*, at higher concentrations (100–12.5 μM), MP activity was suppressed to levels similar to the NC, corresponding to an almost 80% reduction compared to the venom-alone control, with partial recovery observed at lower concentrations (6.25–0.098 μM; Figure 1F,G). In *N. pallida*, prinomastat demonstrated stable inhibition across all tested concentrations (100–0.098 μM), significantly inhibiting MP activity (Figure 1H). Altogether, these findings demonstrate the complete inhibition of PLA_2_ activity by varespladib, the limited and venom-dependent efficacy of marimastat, and the potent, concentration-dependent inhibitory effect of prinomastat on MP activity in these spitting cobra venoms.

### 2.3. Spitting Cobra Venoms Selectively Degrade the α-Chain of Fibrinogen

**Figure 2:**
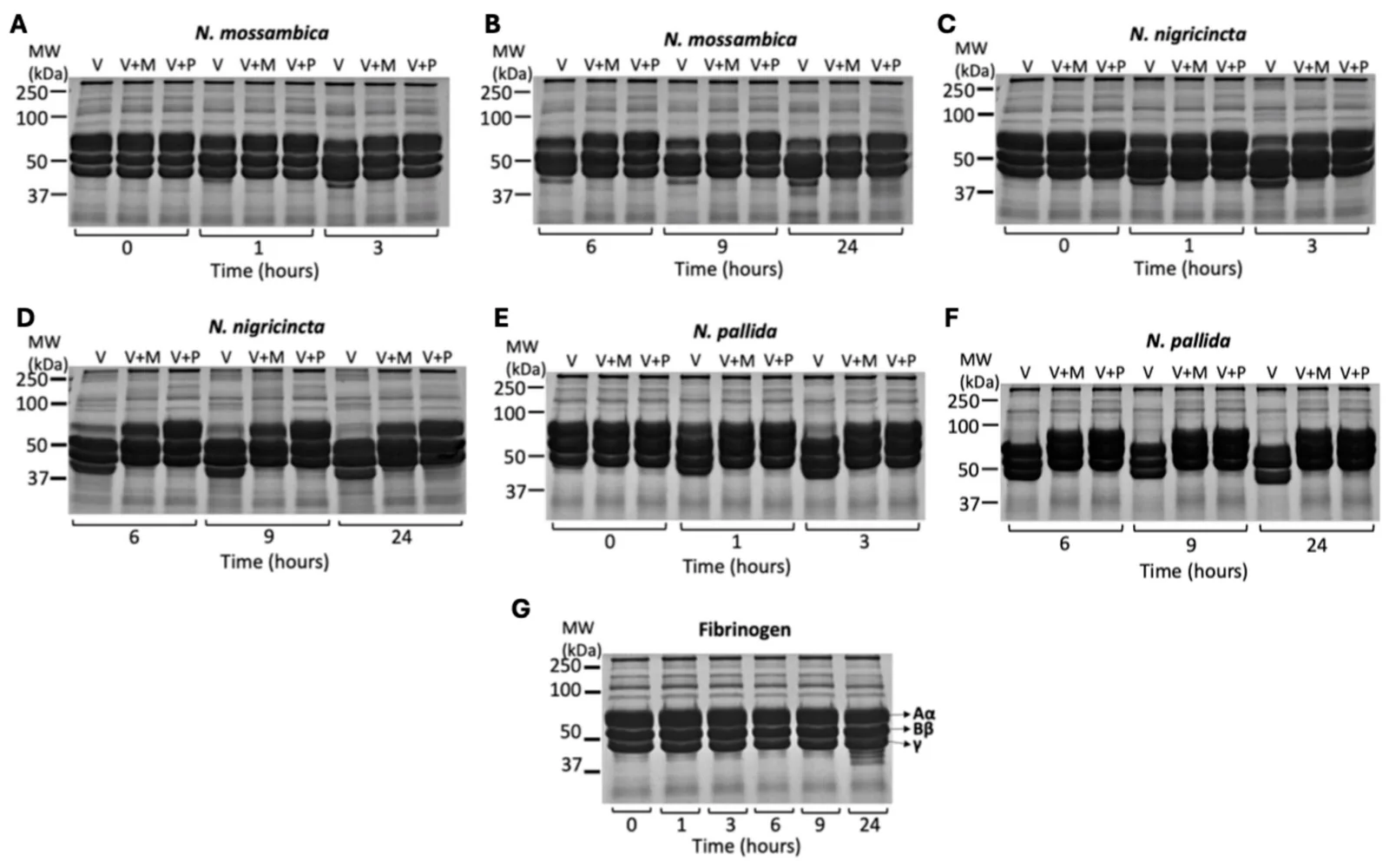
Fibrinogenolytic activity of spitting cobra venoms in the presence and absence of metalloprotease inhibitors. Representative 12% SDS–PAGE gels show the degradation of human fibrinogen (1 mg/mL) after incubation with cobra venoms (100 µg/mL) at 0, 1, 3, 6, 9, and 24 h. Panels (**A**,**B**) correspond to *N. mossambica*; (**C**,**D**) to *N. nigricincta*; and (**E**,**F**) to *N. pallida*. Panel (**G**) shows fibrinogen incubated without venom as a control. Venoms were tested alone (V) or in the presence of marimastat (V+M) or prinomastat (V+P). MW denotes the molecular weight marker.

To assess the fibrinogenolytic activity of the three spitting cobra venoms, human fibrinogen (1 mg/mL) was incubated with each venom (100 μg/mL) either alone (V) or in the presence of marimastat (V+M) or prinomastat (V+P). Samples were collected at different time points (0, 1, 3, 6, 9, and 24 h) and analysed by SDS-PAGE. At baseline (0 h), all fibrinogen chains remained intact in all samples, confirming the absence of spontaneous degradation. In *N. mossambica*, the α-chain degradation was first evident at 1 h and increased progressively, reaching significant levels at 24 h. In contrast, co-incubation with either marimastat or prinomastat prevented α-chain degradation at early time points (1–3 h) (Figure 2A). Although limited degradation occurred at later time points (6–24 h), the α-chain remained visibly more intact than in the venom-alone control, revealing significant inhibition by the inhibitors (Figure 2B). In *N. nigricincta*, venom alone caused progressive α-chain degradation beginning at 1 h, with near-complete degradation by 24 h (Figure 2C). In inhibitor-treated samples, early inhibition of α-chain degradation was evident; however, degradation persisted at later incubation intervals. At 24 h, the α-chain remained detectable in inhibitor-treated samples, whereas it was completely digested in the venom-alone sample, confirming significant inhibition by the inhibitors (Figure 2D). For *N. pallida*, α-chain degradation in the venom-alone samples was rapid, with significant digestion by 3 h and near-complete degradation by 6 h (Figure 2E). Despite this rapid fibrinogenolytic activity, both inhibitors significantly inhibited α-chain degradation at all time points, with residual signal still detectable at 24 h, in contrast to complete degradation in V samples (Figure 2E,F). The fibrinogen-only control confirmed the stability of all three fibrinogen chains (Aα, Bβ, and γ) across incubation periods (Figure 2G). Collectively, these findings show that spitting cobra venoms possess significant, time-dependent fibrinogenolytic activity with strong selectivity for the α-chain, and that both marimastat and prinomastat substantially inhibit this degradation.

### 2.4. Spitting Cobra Venoms Suppress Agonist-Induced Platelet Activation

**Figure 3:**
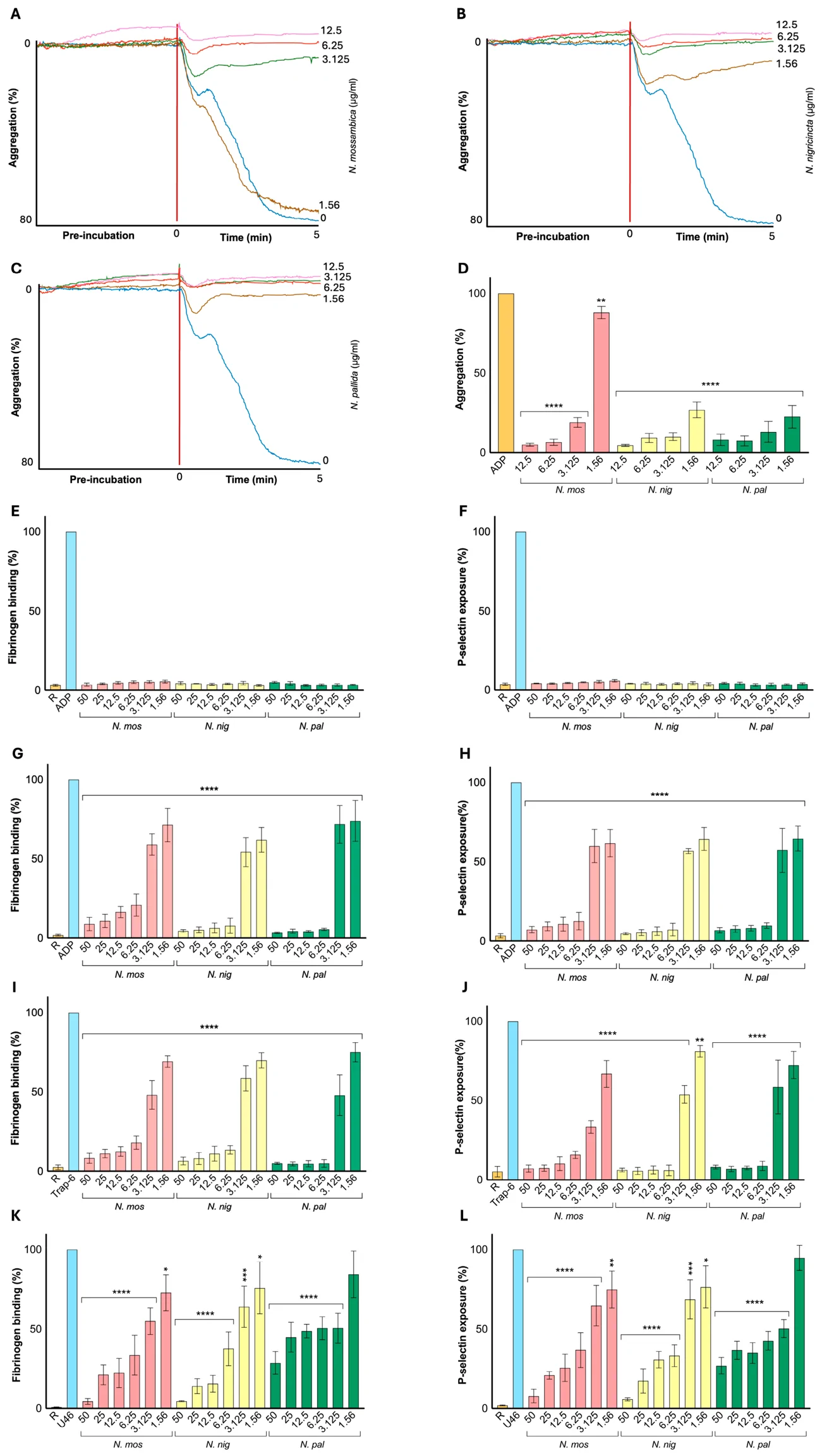
Effects of spitting cobra venoms on platelet activation. (**A**–**D**) Platelet aggregation was measured by optical aggregometry using platelet-rich plasma (PRP) in the presence and absence of various concentrations of venoms upon stimulation with different agonists. Representative aggregation traces are shown for *N. mossambica* (**A**), *N. nigricincta* (**B**), and *N. pallida* (**C**) upon stimulation with ADP (5 µM). (**D**) Quantified data on aggregation responses for all three venoms. Responses were compared with ADP alone (set as 100%). The level of fibrinogen binding following incubation with venoms (50–1.56 µg/mL) with/without different agonists [ADP (5 µM), TRAP-6 (10 µM), or U46619 (4 µM)] is shown in panels (**E**,**G**,**I**,**K**). Similarly, the levels of P-selectin exposure are shown in panels (**F**,**H**,**J**,**L**). The level of activation in resting platelets (R) served as the baseline control. Agonist-only samples (ADP, TRAP-6, or U46619) were set at 100% and used for normalisation. In all graphs, cobra venoms are indicated as *N. mos* (*N. mossambica*), *N. nig* (*N. nigricincta*), and *N. pal* (*N. pallida*). Data are presented as mean ± S.D. (*n* = 4 individual healthy human volunteers). Statistical analysis was performed using one-way ANOVA followed by Tukey’s multiple comparisons test. (* *p* < 0.05, ** *p* < 0.01, *** *p* < 0.001 and **** *p* < 0.0001).

To investigate the effects of spitting cobra venoms on platelet function, optical aggregometry and flow cytometry assays were performed. These assays evaluated platelet aggregation, fibrinogen binding (a marker of inside-out signalling via integrin αIIbβ3 activation), and P-selectin exposure (a marker of α-granule secretion) under resting and agonist-stimulated conditions. During the initial pre-incubation phase of the aggregometry assay, none of the spitting cobra venoms induced platelet aggregation, indicating no direct platelet activation. In contrast, following stimulation with ADP (5 µM), a clear, concentration-dependent inhibitory effect was observed with all three venoms (Figure 3A–D). In *N. mossambica*, aggregation remained close to baseline at higher concentrations (12.5–3.125 μg/mL) but increased markedly at the lowest concentration (1.56 μg/mL), approaching almost 80% of the ADP control (Figure 3A,D). In *N. nigricincta* and *N. pallida*, aggregation remained below 25% of the ADP control across all concentrations (12.5–1.56 μg/mL) (Figure 3B–D).

To further investigate the effects of these venoms on platelet activation pathways, fibrinogen binding and P-selectin exposure were assessed by flow cytometry. To determine whether the venom-induced platelet inhibitory effects were limited to a specific platelet activation pathway or occurred more broadly, platelets were stimulated with various agonists including ADP (stimulates via P_2_Y12 receptor), TRAP-6 (stimulates via PAR-1 and PAR-4 receptors), and U46619 (stimulates via TP receptor), which activate distinct platelet signalling pathways. For all three cobra venoms, samples treated with venom alone did not induce platelet activation under resting conditions (Figure 3E,F). However, upon stimulation with ADP (5 μM) or TRAP-6 (10 μM), all three venoms (50–1.56 μg/mL) produced pronounced, concentration-dependent inhibition of both fibrinogen binding (Figure 3G,I) and P-selectin exposure (Figure 3H,J). At higher venom concentrations (50–6.25 μg/mL), activation markers were suppressed to approximately 5–15% of agonist-control levels. As the venom concentration decreased (3.125–1.56 μg/mL), platelet responses gradually recovered, approaching approximately 60–75% of agonist-induced activation. A similar concentration-dependent inhibition was observed with U46619 (4 μM) as an agonist. Higher venom concentrations (50–12.5 μg/mL) suppressed fibrinogen binding and P-selectin exposure to minimal levels (less than 40%), whereas at lower concentrations (6.25–1.56 μg/mL), activity was gradually restored towards agonist control values (Figure 3K,L). In conclusion, these findings demonstrate that the three spitting cobra venoms cause significant, concentration-dependent inhibition of agonist-induced platelet aggregation and activation, suppressing both integrin αIIbβ3 activation and α-granule secretion pathways.

### 2.5. Fractionation of Spitting Cobra Venoms Reveals Three-Finger Toxins with Platelet-Inhibitory Effects

**Figure 4:**
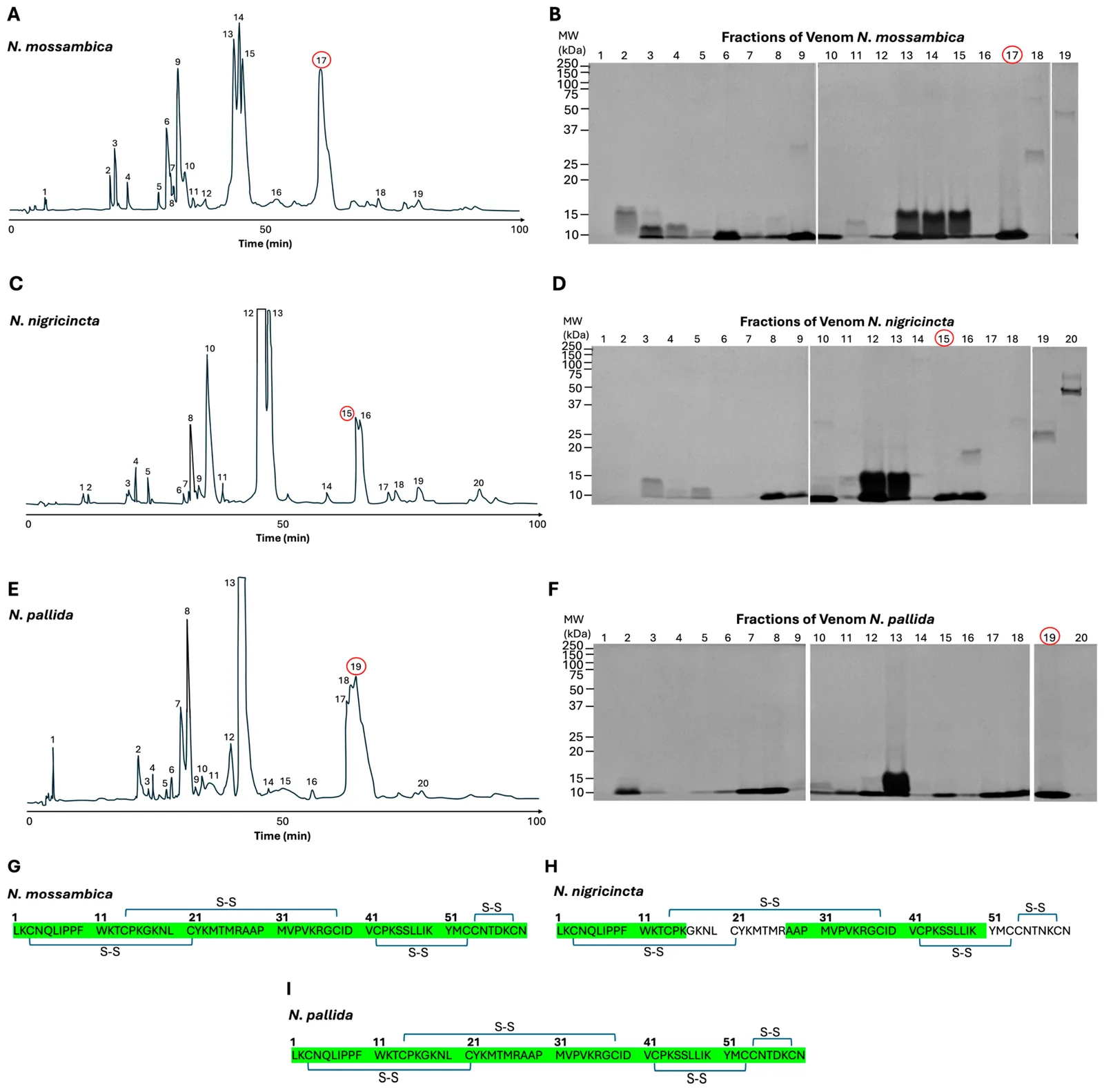
Purification and characterisation of 3FTxs from spitting cobra venoms. Representative RP-HPLC profiles of venoms from *N. mossambica* (**A**), *N. nigricincta* (**C**), and *N. pallida* (**E**) are shown. For each species, 2 mg of lyophilised venom was subjected to C18 RP-HPLC over 100 min, and 19 fractions were collected for *N. mossambica* and 20 for *N. nigricincta* and *N. pallida*. Representative 12% SDS–PAGE gel images show the protein profiles of *N. mossambica* (**B**), *N. nigricincta* (**D**), and *N. pallida* (**F**) of individual fractions. Mass spectrometry analysis of selected purified proteins from the venoms of *N. mossambica* (**G**), *N. nigricincta* (**H**), and *N. pallida* (**I**) identified them as three-finger toxins following tryptic digestion. Green-highlighted regions indicate homology between mass spec-identified sequences and previously reported cytotoxins. Alignments of the *N. mossambica* sequence to a known *N. pallida* cytotoxin, the *N. nigricincta* sequence to a reported *N. mossambica* cytotoxin, and the *N. pallida* sequence to an established *N. pallida* cytotoxin were performed. The identified cytotoxins are shown by red circles (**A**–**F**). MW denotes molecular weight marker.

To identify the major constituents of spitting cobra venoms, 2 mg of each venom was separated by reversed-phase high-performance liquid chromatography (RP-HPLC) on a C18 column using a 100-min gradient. This yielded 19 fractions for *N. mossambica* and 20 fractions each for *N. nigricincta* and *N. pallida*. Chromatographic profiles of all three venoms showed several early-eluting peaks between 15 and 30 min, likely corresponding to relatively hydrophilic components. This was followed by prominent, sharp peaks between 40 and 50 min and additional abundant peaks at approximately 60–70 min, a pattern consistent with hydrophobic, low-molecular-weight three-finger toxin families (Figure 4A,C,E). SDS-PAGE analysis of 20 μL aliquots from each fraction further supported the predominance of low-molecular-weight toxins, with most proteins found within the 10–15 kDa range (Figure 4B,D,F). In all three venoms, late-eluting fractions collected at approximately 60–70 min displayed strong, discrete bands, consistent with relatively enriched toxin populations.

**Figure 5:**
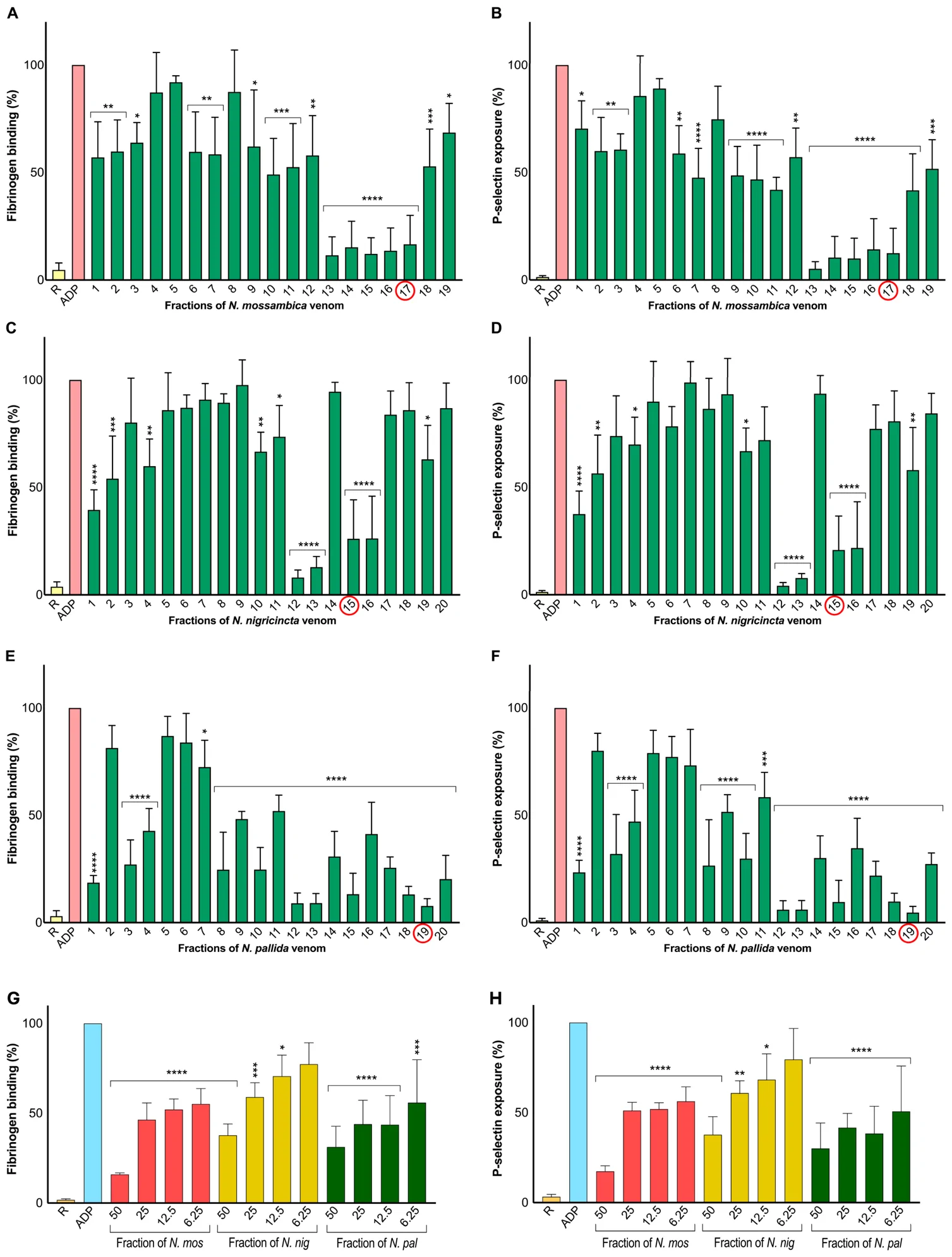
Effects of RP-HPLC fractions and purified toxins from spitting cobra venoms on platelet activation. Fibrinogen binding and P-selectin exposure were measured in all collected RP-HPLC fractions (50 µg/mL) from *N. mossambica* (**A**,**B**), *N. nigricincta* (**C**,**D**), and *N. pallida* (**E**,**F**) venoms. Similarly, the levels of fibrinogen binding (**G**) and P-selectin exposure (**H**) were measured in platelets after treatment with different concentrations of purified toxins (50–6.25 µg/mL) from each spitting cobra venom. In panels (**G**,**H)**, purified cytotoxins from all species are shown in a single graph for clarity; no direct statistical comparisons were made between the toxins. In all panels, platelet activation was normalised to ADP (5 µM), set at 100% and used as the reference control. PRP alone (R) served as the baseline. The identified cytotoxins are shown by red circles (**A**–**F**). (*) Indicates significant differences compared with the ADP control. Data are presented as mean ± SD (*n* = 4). Statistical analysis was performed using one-way ANOVA followed by Tukey’s multiple comparisons test (* *p* ≤ 0.05, ** *p* ≤ 0.01, *** *p* ≤ 0.001, **** *p* ≤ 0.0001).

Flow cytometric screening of all collected fractions (5 μL each) showed that multiple fractions from each spitting cobra venom significantly inhibited ADP-induced fibrinogen binding and P-selectin exposure (Figure 5A–F). However, the magnitude of inhibition of fibrinogen binding and P-selectin exposure varied across fractions. In *N. mossambica*, fraction 17 produced one of the most pronounced inhibitory effects on both activation markers and corresponded to a dominant HPLC peak with a strong, discrete SDS-PAGE band (Figure 4 and Figure 5A,B). In *N. nigricincta*, several fractions showed inhibitory activity, but fraction 15 consistently produced robust suppression, accompanied by a prominent chromatographic peak and a relatively pure protein band (Figure 4 and Figure 5C,D). Similarly, in *N. pallida*, fraction 19 showed strong inhibition and corresponded to a major HPLC peak that exhibited a clear single band on SDS-PAGE (Figure 4 and Figure 5E,F). Based on their abundance, relative purity, and potent platelet-inhibitory activity, these fractions were selected for mass spectrometric characterisation. Peptide mass fingerprinting identified the selected fractions as venom-derived three-finger toxins (3FTxs). The *N. mossambica*-derived fraction showed 100% sequence coverage of a cytotoxin reported from *N. pallida* venom (UniProt accession no. P01468) (Figure 4G). The *N. nigricincta*-derived fraction displayed 65% coverage of a cytotoxin from *N. mossambica* (UniProt accession no. P01467) (Figure 4H). The *N. pallida*-derived fraction showed 100% sequence coverage of a cytotoxin from *N. pallida* venom (UniProt accession no. P01468) (Figure 4I). During mass spectrometry, no other major contaminants were identified in these fractions. Moreover, in gel electrophoresis, these proteins appeared isolated and distinct, although we cannot rule out the possibility of minor contaminants. Hence, although we refer to them as purified toxins in this article, they should be interpreted as three-finger toxin-enriched fractions.

To investigate the impact of the identified purified proteins on platelet activity, flow cytometric assays were performed at different concentrations of each fraction (50–6.25 μg/mL). All three toxins significantly inhibited ADP-induced fibrinogen binding and P-selectin exposure in a concentration-dependent manner (Figure 5G,H). At the highest concentration, the toxin from *N. mossambica* venom reduced platelet activation to approximately 10% compared to the control, with partial recovery (around 50%) at lower concentrations (25–6.25 μg/mL). The toxin from *N. nigricincta* venom exhibited moderate inhibition (around 40–60%) at higher concentrations (50–25 μg/mL), followed by a gradual restoration of activity to around 70–80% as the concentration decreased (12.5–6.25 μg/mL). The toxin from *N. pallida* venom similarly suppressed platelet activation potently at the highest concentration (50 μg/mL), with dose-dependent recovery (around 50–60%) at lower concentrations (25–6.25 μg/mL). In conclusion, these findings demonstrate that spitting cobra venoms are enriched in three-finger toxins, which play a significant role in inhibiting platelet reactivity.

### 2.6. Spitting Cobra Venoms and Their Purified Toxins Disrupt Blood Coagulation

**Figure 6:**
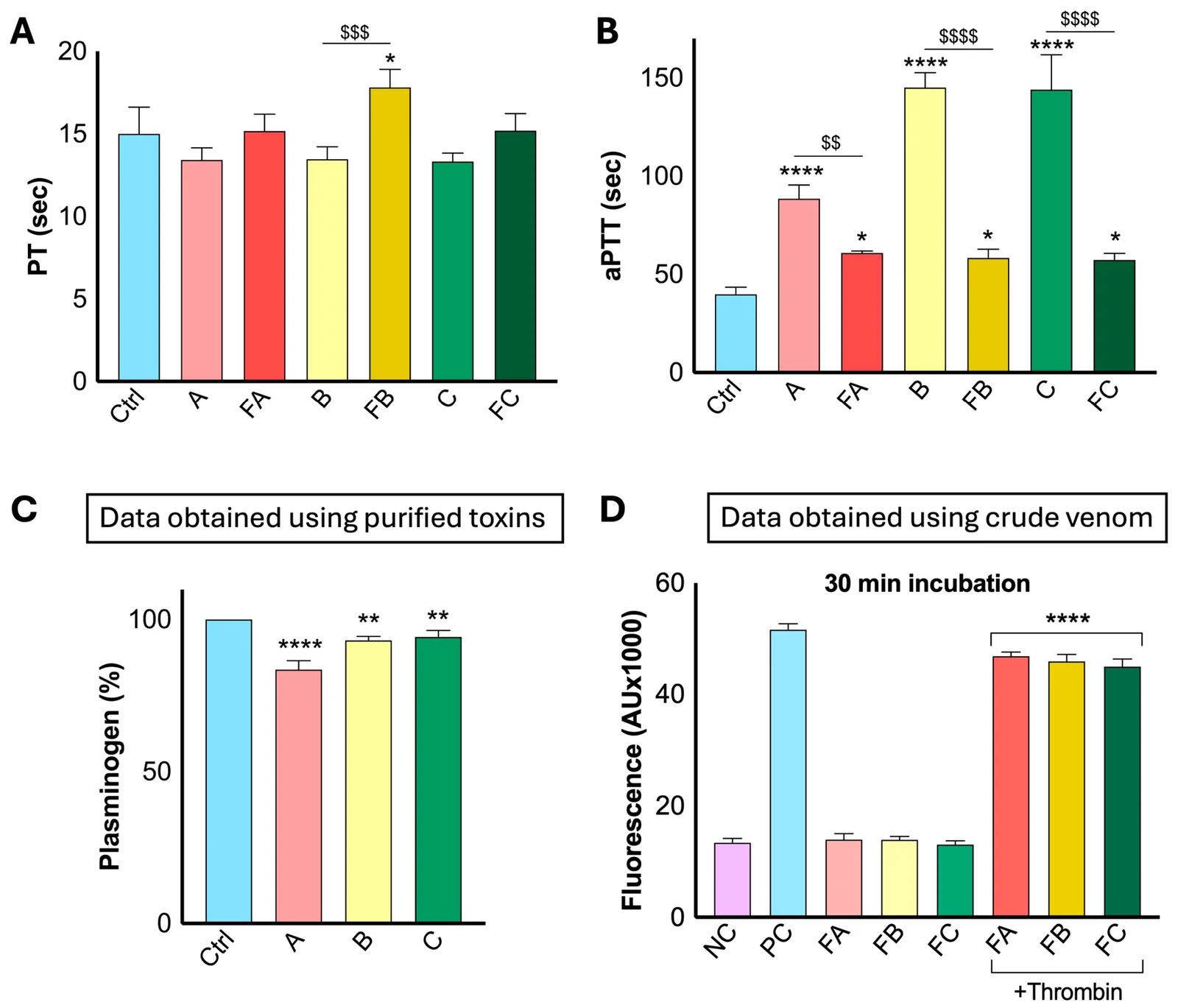
Effects of spitting cobras and their purified toxins on coagulation activity. (**A**) Prothrombin time (PT), (**B**) activated partial thromboplastin time (aPTT), and (**C**) plasminogen activity were measured in citrated human plasma with/without spitting cobra venoms and their purified proteins (only (**C**)) using an automated coagulation analyser. (**D**) Thrombin activity was assessed using a fluorogenic substrate (BAAMC) after incubating purified proteins (50 µg/mL each) with thrombin (5 units/mL). In all graphs, venoms are shown as A (*N. mossambica*), B (*N. nigricincta*), and C (*N. pallida*), and purified fractions as FA, FB, and FC, in the same order. The venoms and toxins were used at concentrations of 1.56 µg/mL (**A**,**B**) and 50 µg/mL (**C**,**D**). For panels (**A**,**B**), data are presented as mean ± S.D. (*n* = 4), and two-way ANOVA along with Tukey’s multiple comparisons test was performed to compare whole venom and purified toxins with the control and then compare purified toxins with their corresponding whole venoms. For panels (**C**) (*n* = 4) and (**D**) (*n* = 6), data are presented as mean ± S.D., and statistical analysis was performed using one-way ANOVA with Tukey’s multiple comparisons test. (*) indicates significant differences compared with control, and ($) indicates significant differences between fractions and their respective whole venoms. In panel (**D**), (*) denotes significant differences between each thrombin-treated fraction and its respective fraction alone (* *p* ≤ 0.05, ** *p* ≤ 0.01, and **** *p* ≤ 0.0001) ($$ *p* ≤ 0.01, $$$ *p* ≤ 0.001, and $$$$ *p* ≤ 0.0001).

To evaluate the impact of the three spitting cobra venoms (1.56 μg/mL) and their purified toxins (1.56 μg/mL) on coagulation pathways, prothrombin time (PT) and activated partial thromboplastin time (aPTT) assays were performed to assess the extrinsic and intrinsic pathways, respectively. In the PT assay, all venoms induced a modest but non-significant shortening of clotting time, reducing PT by around 15% relative to the control (Figure 6A). This indicates a mild but non-significant procoagulant effect on the extrinsic pathway. In contrast, purified toxin fractions did not display this procoagulant effect. Fractions from *N. mossambica* and *N. pallida* restored PT to control levels, indicating loss of procoagulant activity. Notably, the *N. nigricincta* fraction extended PT beyond control values by approximately 15%, suggesting a mild anticoagulant shift relative to both the control and its corresponding whole venom (Figure 6A). The aPTT assay revealed significant disruption of the intrinsic pathway by whole venoms (Figure 6B). *N. mossambica* prolonged clotting time to more than double the control value, while *N. nigricincta* and *N. pallida* extended aPTT to nearly threefold above baseline, indicating strong anticoagulant activity. Purified toxin fractions also prolonged aPTT by approximately 40% relative to the control. However, their effects were significantly weaker than those of the corresponding whole venoms in aPTT, suggesting that the inhibition of intrinsic coagulation is not attributable to these toxins alone and reflects the combined actions of multiple venom components.

To assess plasminogen activity, a chromogenic plasminogen assay kit was used in a Ceveron T100 coagulation analyser with citrated human plasma. Whole venoms (50 μg/mL) reduced plasminogen activity by approximately 10–20% relative to the control, indicating mild but statistically significant suppression of fibrinolytic capacity (Figure 6C). Thrombin activity was further evaluated using a fluorogenic substrate (BAAMC). Purified toxins (50 μg/mL each) were incubated with thrombin for 30 min; the substrate was then added, and fluorescence was measured using spectrofluorimetry. The fractions alone showed no activity towards the thrombin substrate; however, upon thrombin addition, they reduced thrombin activity by approximately 10–15% relative to the positive control (PC), indicating modest but significant inhibitory effects (Figure 6D). Together, these findings reveal that spitting cobra venoms largely impair intrinsic coagulation, whereas purified cytotoxins contribute, in part, through moderate anticoagulant and thrombin-inhibitory effects. This suggests the presence of additional components that contribute to the anticoagulant activities of these venoms.

### 2.7. Spitting Cobra Venoms and Their Isolated Proteins Impair INTEM Activity

**Figure 7:**
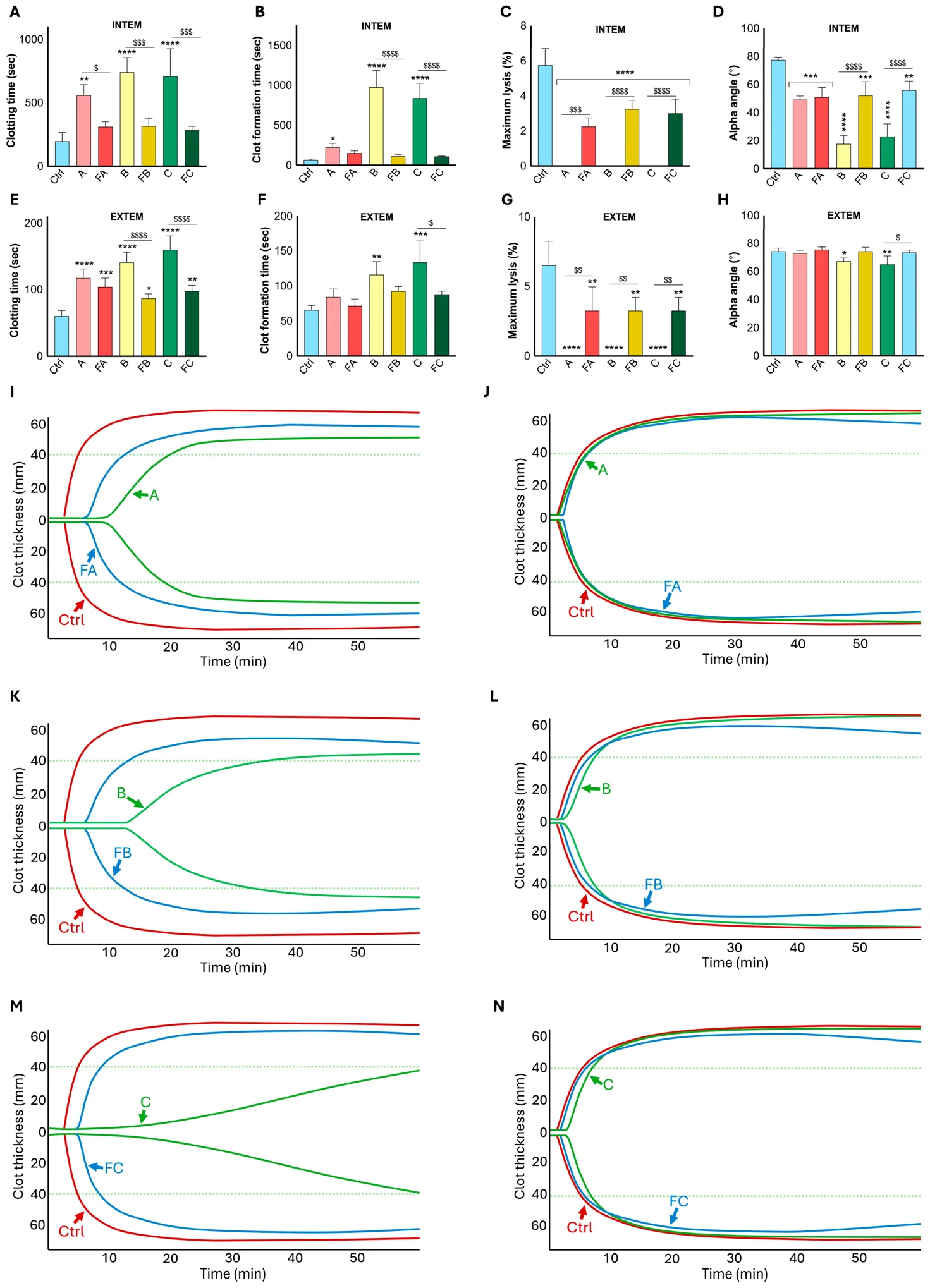
Effects of spitting cobras and their purified cytotoxin fractions on blood clotting. (**A**–**N**) ROTEM analysis of the INTEM and EXTEM pathways. Clotting time (CT) is shown in (**A**) (INTEM) and (**E**) (EXTEM); clot formation time (CFT) in (**B**) (INTEM) and (**F**) (EXTEM); maximum lysis (ML) in (**C**) (INTEM) and (**G**) (EXTEM); and alpha angle (α-angle) in (**D**) (INTEM) and (**H**) (EXTEM). (**I**–**N**) Representative ROTEM traces. INTEM traces are shown in (**I**,**K**,**M**), and EXTEM traces in (**J**,**L**,**N**). Panels (**I**,**J**) correspond to *N. mossambica*, (**K**,**L**) to *N. nigricincta*, and (**M**,**N**) to *N. pallida*. In all graphs, venoms are indicated as A (*N. mossambica*), B (*N. nigricincta*), and C (*N. pallida*), and purified fractions as FA, FB, and FC, respectively. PBS served as the control (Ctrl) in all assays. Whole venoms and purified fractions were tested at 1.56 µg/mL. For panels (**A**–**H**), data are presented as mean ± SEM (*n* = 4), and two-way ANOVA with Tukey’s multiple comparisons test was performed to compare the whole venom and fractions with the control and the purified fraction with its corresponding whole venom. (*) indicates significant differences compared with the control, and ($) indicates significant differences between fractions and their respective whole venoms (* *p* ≤ 0.05, ** *p* ≤ 0.01, *** *p* ≤ 0.001 and **** *p* ≤ 0.0001) ($ *p* ≤ 0.05, $$ *p* ≤ 0.01, $$$ *p* ≤ 0.001 and $$$$ *p* ≤ 0.0001).

To assess the impact of spitting cobra venoms (1.56 μg/mL each) and their purified toxins (1.56 μg/mL each) on whole blood clotting, rotational thromboelastometry (ROTEM) analysis was performed using INTEM (for the intrinsic pathway) and EXTEM (for the extrinsic pathway) assays. In the INTEM assay, whole venoms produced significant inhibition of intrinsic coagulation (Figure 7A–D; representative traces are shown in I, K & M). All three whole venoms significantly prolonged clotting time, increasing it to approximately three times the control value in *N. mossambica* and nearly four times in *N. nigricincta* and *N. pallida*. In contrast, purified toxins produced only modest prolongation of clotting times, approximately 40% above control, and remained significantly lower than their corresponding whole venoms (Figure 7A). A similar pattern was observed for clot formation time, with all whole venoms significantly prolonging it (Figure 7B). *N. nigricincta* and *N. pallida* increased clot formation time by almost tenfold compared to the control, while *N. mossambica* produced only a mild but significant extension. Toxins from all species produced only minimal extensions relative to the control (approximately 2-fold over control), and all were significantly lower than the effects observed with their corresponding whole venoms (Figure 7B). For maximum lysis, all whole venoms abolished fibrinolysis, reducing lysis by approximately 100% compared with control. Toxins partially restored lysis to almost one-third (*N. mossambica*) and to one-half (*N. nigricincta* and *N. pallida*) of baseline levels, significantly higher than their corresponding whole venoms but still significantly reduced compared to the control (Figure 7C). Consistent with these findings, whole venoms markedly reduced the alpha angle, indicating impaired clot propagation. *N. mossambica* reduced the alpha angle by approximately 35%, while *N. nigricincta* and *N. pallida* caused reductions of nearly 50–60%, showing significant extension of clot kinetics. Toxins produced milder reductions (around 30% below control), and in *N. nigricincta* and *N. pallida*, they significantly improved the alpha angle compared to their corresponding whole venom (Figure 7D).

In the EXTEM assay, the effects of whole venoms were more moderate but remained significant across several parameters. Whole venoms significantly prolonged clotting time in all three species, with approximately a two-fold increase for *N. mossambica*, a 1.5-fold increase for *N. nigricincta*, and up to a threefold increase for *N. pallida* relative to control (Figure 7E; traces are shown in J, L & N). Purified toxins from all three species also significantly extended clotting time, albeit to a lesser extent than their corresponding whole venoms. The toxin from *N. mossambica* reproduced roughly 90% of its corresponding whole venom activity, whereas the purified proteins from *N. nigricincta* and *N. pallida* venoms reproduced approximately 65% of their respective whole venom effects, indicating a significant reduction in anticoagulant potency for the latter two species. For clot formation time, *N. nigricincta* and *N. pallida* venoms approximately doubled the clot formation time compared to the control, whereas *N. mossambica* caused minimal, nonsignificant changes (Figure 7F). The purified toxins from *N. mossambica* and *N. pallida* produced values comparable to the control, with the *N. pallida* toxin being significantly lower than its corresponding whole venom. In contrast, the *N. nigricincta* toxin induced a modest but statistically significant prolongation relative to the control, although this effect remained significantly weaker than that of the corresponding whole venom. All venoms reduced maximum lysis to zero, representing complete and significant suppression of fibrinolysis compared to the control (Figure 7G). Purified toxins partially restored lysis to approximately 60% of control levels, which were significantly higher than those of their corresponding whole venoms. For the α-angle, whole venoms of *N. nigricincta* and *N. pallida* significantly reduced clot propagation by approximately 15%, while *N. mossambica* had no significant effect compared to the control (Figure 7H). None of the purified toxins significantly altered the α-angle compared to the control. However, purified toxins from *N. nigricincta* and *N. pallida* showed significantly higher activity than their respective whole venoms, indicating partial recovery of clot propagation relative to the whole venoms. Collectively, these findings show that whole spitting cobra venoms exert significantly potent anticoagulant effects, particularly within the intrinsic pathway. Meanwhile, the purified toxins contribute only partially to these effects. This suggests that these toxins contribute to venom-induced coagulopathy but are insufficient to account for the complete anticoagulant phenotype, which likely depends on additional venom components acting independently or synergistically.

### 2.8. Spitting Cobra Venoms and Their Toxins Induce Haemolysis

**Figure 8:**
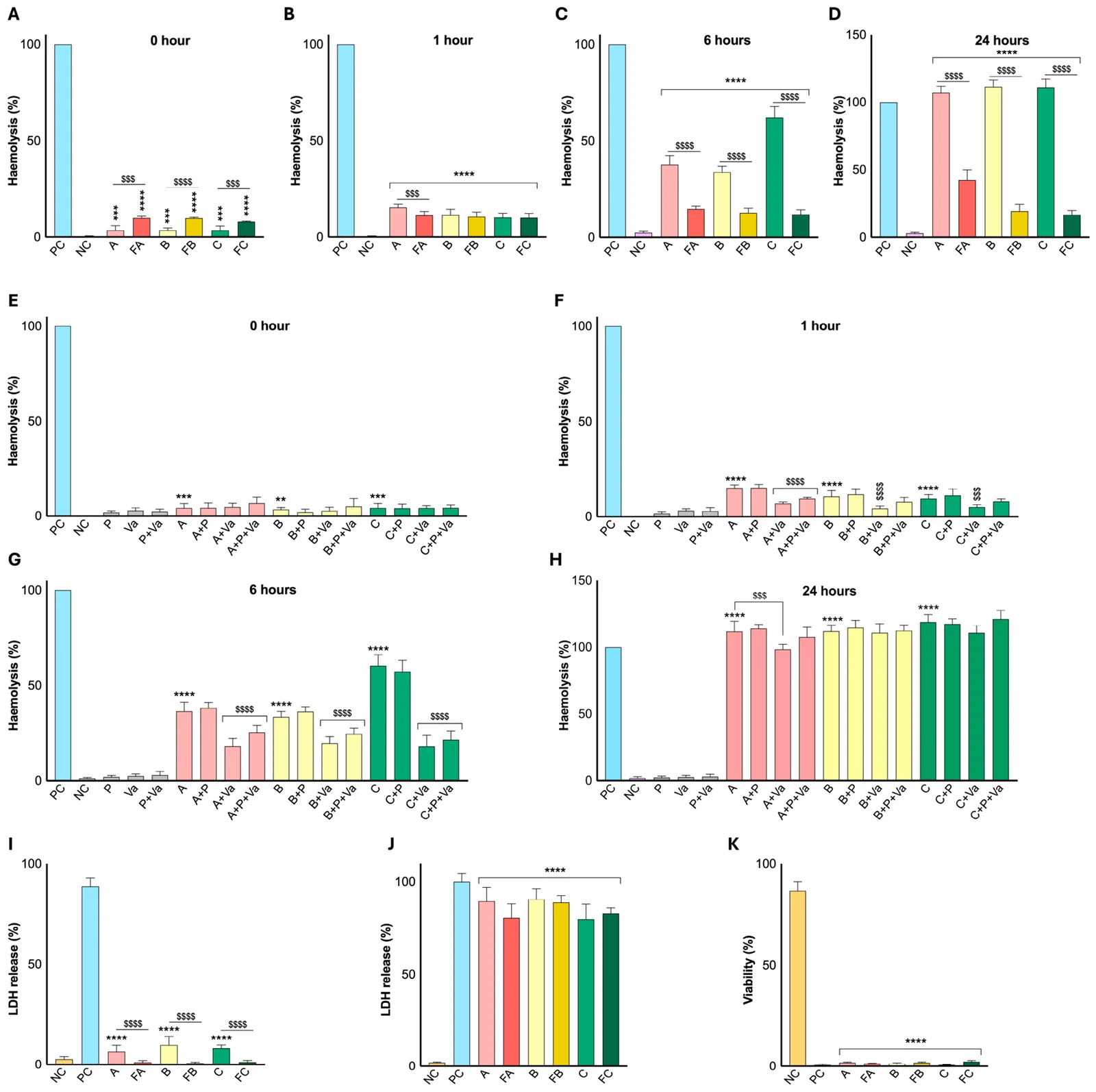
Cytotoxic effects of spitting cobra venoms and their purified toxins. Haemolytic activity of whole venoms (50 µg/mL) and their corresponding purified toxins (50 µg/mL) was assessed using isolated human erythrocytes incubated for 0 h (**A**), 1 h (**B**), 6 h (**C**), and 24 h (**D**). Haemolytic activity of whole venoms (50 µg/mL) was also analysed in the presence or absence of varespladib (Va), prinomastat (P), and both (P+Va; 100 µM each), and measured at 0 h (**E**), 1 h (**F**), 6 h (**G**), and 24 h (**H**). (**I**) LDH release from isolated human platelets treated with whole venoms or purified toxins (50 µg/mL) was measured using the CyQUANT™ LDH Cytotoxicity kit. (**J**) LDH release from AB1190 undifferentiated human myoblast cells following treatment with venoms or purified toxins (50 µg/mL) was assessed using the same kit. (**K**) Viability of AB1190 myoblasts treated with venoms or purified toxins (50 µg/mL) was assessed using the MTS assay. PBS (**A**–**I**) and fresh growth media (**J**,**K**) served as the negative control (NC). Triton X-100 (1%; Sigma, UK) was used as the positive control (PC) for all haemolytic assays. Results are expressed as a percentage relative to the PC for panels (**A**–**J**), and to the NC for panel (**K**). Absorbance was recorded at 540 nm in (**A**–**H**) and 490 nm in (**I**–**K**) using a plate reader. Data are presented as mean ± S.D. (*n* = 4). Statistical analysis was performed using two-way ANOVA followed by Tukey’s test. (*) indicates significant differences compared with the NC, and ($) indicates significant differences between inhibitor-treated samples or purified fractions and their respective whole venoms (** *p* < 0.01, *** *p* < 0.001 and **** *p* < 0.0001) ($$$ *p* ≤ 0.001 and $$$$ *p* ≤ 0.0001).

To measure the impact of spitting cobra venoms on haemolytic activity, isolated human erythrocytes were incubated with the venoms (50 μg/mL each) and their purified toxins (50 μg/mL). At early time points (0–1 h), whole venoms produced low but significant haemolysis (around 5–20%), whereas the toxins induced similar or slightly greater lysis (Figure 8A,B). At 6 h, venoms showed significantly higher haemolysis, reaching around 40% for *N. mossambica* and *N. nigricincta* and around 70% for *N. pallida*. In contrast, the haemolytic activity of the purified toxins remained limited to approximately 20%, indicating a significant reduction relative to their corresponding whole venoms (Figure 8C). At 24 h, venoms caused near-complete haemolysis (almost 100%), whereas the purified proteins remained substantially lower (20–40%), compared with their corresponding venoms (Figure 8D). These findings indicate that purified toxins contribute to erythrocyte lysis, but at a lower level than whole venoms, suggesting that additional components are likely involved in the overall haemolytic activity of venoms.

To investigate the effects of metalloproteases and PLA_2_ enzymes in whole venoms on haemolysis, prinomastat (P, SVMP inhibitor; 100 µM), varespladib (Va, PLA_2_ inhibitor; 100 µM), and their combination (P+Va) were tested in this assay. At baseline (0 h), no significant changes in the haemolytic activity were observed (Figure 8E). Across all time points and in all three venoms, prinomastat alone failed to inhibit haemolysis, as the inhibitor-treated samples remained comparable to the corresponding whole venoms, indicating minimal involvement of metalloproteases in erythrocyte lysis. However, at 1 h, varespladib significantly reduced haemolysis in all cobra venoms by approximately 40–60% relative to their corresponding venom alone (Figure 8F). The combined treatment with prinomastat and varespladib showed partial inhibition at 1 h and reached significance only in *N. mossambica*. At 6 h, varespladib continued to markedly suppress haemolysis in all species, reducing activity by roughly half in *N. mossambica* and *N. nigricincta* and by around 70% in *N. pallida* venom (Figure 8G). The combination of prinomastat and varespladib treatment similarly produced significant inhibition across all species, with reductions of around 20% in *N. nigricincta* and *N. mossambica*, and around 50% in *N. pallida*. However, by 24 h, inhibitory effects diminished significantly; varespladib reduced haemolysis only slightly but significantly in *N. mossambica*, whereas in *N. nigricincta* and *N. pallida*, the reduction was not significant (Figure 8H). These data demonstrate that varespladib-mediated PLA_2_ inhibition significantly prevented venom-induced haemolysis at earlier time points, whereas metalloprotease inhibition had little effect, indicating that the haemolytic activity of these venoms may be solely due to PLA_2_, with or without minor contributions from other venom components.

### 2.9. Spitting Cobra Venoms Induce a Mild Cytotoxicity Effect on Platelets

To investigate the cytotoxic effects of spitting cobra venoms and their purified toxins (50 µg/mL each) on platelets, a lactate dehydrogenase (LDH) release assay was performed on isolated human platelets. Whole venoms induced mild but significant cytotoxicity (around 10%) relative to the negative control (NC), whereas the purified toxins did not induce any LDH release (Figure 8I). Furthermore, to assess myotoxicity, AB1190 myoblasts were treated with whole venoms or purified toxins (50 µg/mL), and cytotoxicity was quantified by LDH release. Both whole venoms and their toxins induced extensive membrane damage, with approximately 80–90% LDH release, indicating severe cytotoxicity (Figure 8J). To further confirm these findings, AB1190 cell viability was also assessed using an MTS assay with/without venoms and purified toxins. Both whole venoms and their purified toxins significantly reduced metabolic activity, similar to the NC, confirming a significant loss of viability (Figure 8K). Together, these findings show that spitting cobra venoms induce potent myotoxic effects, while having only a minimal impact on platelets. The purified toxins appear to play a major role in myotoxicity, indicating that these isolated 3FTxs may have a specific role in inducing cytotoxicity to specific cell types.

## 3. Discussion

Research on the haemostatic effects of snake venoms has focused predominantly on viper venoms, while the haemotoxicity of elapid venoms has been comparatively understudied, despite extensive study of their neurotoxic and cytotoxic effects [17]. Consequently, although the haemostatic effects of some cobra species have previously been investigated, the effects of African spitting cobra venoms on blood and haemostasis remain only partially characterised [34,35]. This gap is clinically important, as spitting cobra venoms account for a substantial proportion of snakebite morbidity in sub-Saharan Africa and are strongly associated with severe tissue injury [4]. Recent studies have begun to reveal a more distinctive haemostatic profile for some of these species, indicating an anticoagulant phenotype that distinguishes them from the typical elapid venom neurotoxic and cytotoxic activities [7]. These findings are clinically important, as venoms from *N. mossambica*, *N. nigricincta,* and *N. pallida* cause severe local tissue damage, which may be exacerbated by anticoagulant effects that impair local wound repair, thereby promoting necrosis [7,17]. This may also explain the limited efficacy of current antivenoms against dermonecrosis induced by spitting cobra venoms and highlight the importance of mechanism-driven toxin profiling in these venoms [8].

Consistent with this interpretation, our enzymatic profiling revealed significant PLA_2_ and metalloprotease activities in the venoms of *N. mossambica*, *N. nigricincta,* and *N. pallida*. These findings align with published proteomes of *N. mossambica*, *N. nigricincta* and *N. pallida*, in which three-finger toxins (3FTxs) predominate (50–70%), whereas PLA_2_s (30–40%) and metalloproteases (<5%) are consistently present as secondary components [4,36,37]. Similar patterns have been reported in other elapid venoms, where low abundances of metalloproteases (2.8%) and serine proteases (0.11%) coexist with a predominance of 3FTxs (52%) and PLA_2_ (27%), supporting their likely contribution to cytotoxicity and anticoagulant effects [38,39,40,41]. In contrast, viper venoms typically contain higher abundances of metalloproteases, serine proteases, and PLA_2_s, which underlie their characteristic haemorrhagic phenotype through extensive proteolytic degradation of vascular and coagulation components, as well as membrane damage [41,42]. All tested spitting cobra venoms also selectively degraded the fibrinogen α-chain, and this effect was markedly inhibited by both metalloprotease inhibitors, indicating that fibrinogenolysis is mediated primarily by metalloproteases. This supports the view that, although present at lower abundance, metalloproteases remain functionally important components of these spitting cobra venoms [4,36]. Similar fibrinogenolytic activity has been previously reported for spitting cobra venoms such as *N. nigricollis*, *N. mossambica*, *N. nigricincta*, and *N. pallida*, as well as other members of the *Hemachatus*/*Naja* genus. These venoms typically degrade the fibrinogen Aα-chain while sparing the Bβ- and γ-chains, whereas *Ophiophagus* venoms cleave both the Aα- and Bβ-chains [43]. Viper venoms, particularly those of *Crotalus*, *Echis*, *Macrovipera*, and several pit vipers, often exhibit more extensive fibrinogenolytic activity mediated by higher metalloprotease abundance, with rapid degradation of the Aα-chain and frequent cleavage of the Bβ-chain, resulting in more pronounced haemorrhagic effects and defibrination [44,45,46].

Platelet dysfunction is another major mechanism for venom-induced haemostatic disturbances. Our findings reveal that whole venoms of *N. mossambica*, *N. nigricincta*, and *N. pallida* significantly impair platelet activity. The venoms caused pronounced inhibition of ADP-induced platelet aggregation, fibrinogen binding and P-selectin exposure, with minimal LDH release from platelets. This pattern indicates functional impact on platelet activation rather than significant cytotoxic effects on platelets under the conditions tested. Similar platelet-modulating effects have been described for other cobra venoms, including *N. nigricollis*, *N. naja*, *N. haje*, and *N. melanoleuca*, as well as individual toxins such as mocarhagin. These data illustrate that both metalloproteases and PLA_2_s can disrupt key adhesive and activation pathways in platelets [47,48,49,50]. In viper venoms, modulation of platelet activation is more diverse, with toxins that either inhibit or activate platelets, including disintegrins such as echistatin and contortrostatin that target integrin-mediated fibrinogen binding [51,52,53,54]. The inhibition of platelet function observed in our study may therefore contribute to the haemorrhagic manifestations often reported after cobra envenomings. Notably, bites from *N. nigricollis* have been associated with spontaneous haemorrhage and defective clot retraction, linked to platelet dysfunction [55], supporting the view that impaired platelet activation may contribute to systemic haemostatic disturbance in at least a subset of cases of cobra envenoming. Notably, three independent platelet agonists (ADP, TRAP-6 and U46619) used in our study demonstrate that the spitting cobra venom-induced inhibitory effects are not restricted to a single platelet activation pathway, suggesting a broad disruption of platelet signalling and functions.

The blood coagulation assays also support a predominantly anticoagulant phenotype, with a stronger effect on the intrinsic than the extrinsic pathway for spitting cobra venoms. PT was only modestly altered, whereas aPTT was significantly prolonged. ROTEM further demonstrated delayed clot initiation and propagation via the intrinsic pathway, consistent with impaired fibrin formation and reduced clot stabilisation. The accompanying reduction in plasmin activity and mild inhibition of thrombin activity suggest effects on fibrinolytic balance and thrombin-dependent clot stabilisation. Taken together, these findings are most consistent with disruption of intrinsic coagulation and downstream clot formation, with possible secondary involvement of the common pathway of blood clotting. These findings are consistent with previous thrombelastography (TEG) studies, which also reported predominantly anticoagulant effects in African spitting cobras, including *N. nigricollis* and *N. nubiae*, as well as other cobra species such as *N. kaouthia*, *N. haje*, *N. samarensis*, *N. siamensis*, and *N. sputatrix*. Similar to these studies [34,35], our ROTEM analyses demonstrated impaired clot formation and delayed clot development. Another recent study of African spitting cobra venoms demonstrated potent inhibition of the prothrombinase complex, with stronger effects on factor Xa than on thrombin. The anticoagulant effect of *N. nigricollis* venom was attributed primarily to PLA_2_s rather than metalloproteases [17,29]. Related anticoagulant effects have also been reported for *N. kaouthia*, *N. atra*, and *Pseudechis* species [56,57,58]. Although *N. nigricincta nigricincta* has also been reported to exhibit procoagulant and thrombolytic activities [18], the overall pattern in our study favours an anticoagulant effect. This contrasts with many viper venoms, in which abundant metalloproteases and serine proteinases drive procoagulant or consumption coagulopathic states by activating factors such as prothrombin and factor X [51]. Clinical reports of cobra and other elapid bites involving prolonged clotting times and systemic bleeding are therefore consistent with the haemostatic profile observed here [10].

Following reverse-phase fractionation of *N. mossambica*, *N. nigricincta*, and *N. pallida* venoms, one abundant, highly purified protein with platelet-inhibitory effects from each venom was selected for further analysis. Mass spectrometry identified these fractions as cytotoxins belonging to the 3FTx family. These toxins are small, disulphide-rich, non-enzymatic proteins that dominate elapid venoms and are especially abundant in African spitting cobras [36,59,60,61]. Although traditionally associated with membrane disruption and cytotoxicity [21,38,62,63], accumulating evidence suggests that the 3FTxs also possess haemotoxic activities [64,65,66]. In this study, purified toxins demonstrated several major effects of the corresponding whole venoms, specifically inhibition of platelet activation, as evidenced by reduced fibrinogen binding and P-selectin exposure. They also exhibited partial haemolysis and marked cytotoxicity towards muscle cells, while causing minimal cytotoxicity to platelets, indicating selective cellular activity rather than broad cellular destruction. In contrast, these proteins displayed only part of the anticoagulant phenotype observed with whole venoms, implying that broader haemostatic disruption arises from cooperative or additive effects involving additional toxin classes. This interpretation is consistent with prior studies of purified cobra toxins, including KT-6.9 from *N. kaouthia*, a platelet-inhibitory 3FTx, cytotoxins from *N. nigricollis* with antiplatelet and anticoagulant properties and the *Hemachatus haemachatus* 3FTxs such as ringhalectin and exactin, which interfere with factor X activation and fibrin clot formation [24,47,64,66,67]. Collectively, these findings support a model in which 3FTxs contribute substantially to platelet dysfunction, whereas the full anticoagulant phenotype of the whole venoms reflects synergistic effects of multiple toxins.

Although African spitting cobra venoms are best known for their cytotoxic effects [36,68], the present findings show that venoms from *N. mossambica*, *N. nigricincta*, and *N. pallida* also inhibit platelet activation and disrupt the coagulation cascade. The purified 3FTxs contributed to the antiplatelet activity and reproduced part of the anticoagulant phenotype observed in whole venoms. From a translational perspective, 3FTxs are attractive scaffolds for protein engineering and bioprospecting because they are small, non-enzymatic, and disulphide-rich, with a rigid and stable structural framework [61,69]. The ability of purified toxins to modulate haemostasis offers insight into their potential development as therapeutic leads for thrombotic conditions. This concept is supported by existing drugs, such as eptifibatide and tirofiban, which were derived from snake-venom disintegrins and are widely used as antiplatelet agents [70,71,72]. Similarly, batroxobin, a thrombin-like enzyme from *Bothrops* venom, has been developed to control fibrinogen levels in both bleeding and thrombotic conditions [73,74]. Nevertheless, the safety of 3FTxs requires careful evaluation, and structural modifications will be needed to mitigate their inherent cytotoxicity prior to therapeutic use [36,68]. While the haemotoxic activities observed in this study were not extensive, they provide insight into the mechanisms underlying platelet dysfunction and coagulation impairment during envenomings from these snake species. Based on our findings, purified toxins likely exert their effects through non-enzymatic interactions with cell membranes or platelet surface receptors, leading to impaired platelet activation, disruption of the coagulation cascade, and cytotoxic effects on muscle cells.

Beyond improving mechanistic understanding, our enzymatic inhibition data have direct translational implications for snakebite therapeutics. Varespladib (LY315920) abolished PLA_2_ activity across all three venoms and significantly attenuated early haemolysis, whereas prinomastat produced concentration-dependent inhibition of metalloprotease activity and prevented fibrinogenolysis. These observations align with a growing body of preclinical work demonstrating that repurposed small-molecule enzyme inhibitors can neutralise key toxic activities of African elapid venoms, offering broader coverage than conventional antivenoms [7,75]. Notably, varespladib has progressed to clinical evaluation for snakebite envenoming through the BRAVO trial, the first Phase II study of an oral small-molecule inhibitor for this indication, with excellent tolerability and pharmacokinetic data [76]. Combination strategies pairing PLA_2_ and metalloprotease inhibitors have been shown to act synergistically in vivo against viper venoms, raising lethality endpoints and reducing dermonecrotic injury [77,78]. The complementary action profiles observed here, i.e., varespladib limiting PLA_2_-mediated haemolysis and prinomastat blocking metalloprotease-driven fibrinogenolysis, reinforce the rationale for multi-target inhibitor combinations as adjuncts or, potentially, as field-deployable interventions that could be administered prior to hospital arrival following snakebites.

From a public health perspective, the haemostatic and cytotoxic activities described here are particularly relevant to rural and agricultural settings, where most spitting cobra envenomings occur. Snakebite was reinstated to the World Health Organisation (WHO) list of priority neglected tropical diseases in 2017, with a global target to halve snakebite-related deaths and disabilities by 2030 [79]. Achieving this in sub-Saharan Africa, where *N. mossambica*, *N. nigricincta* and *N. pallida* are commonly responsible for envenoming, will require interventions that address the well-documented limitations of cold-chain-dependent intravenous antivenoms, including delayed access, anaphylactoid reactions and limited efficacy against local tissue damage [7,33,80,81,82]. The progressive haemolysis and severe myotoxicity demonstrated here by purified toxins underscore why early intervention matters. Cellular and haemostatic damage accumulate within the first hours after envenoming, often before the patient reaches a facility capable of administering antivenom. Therefore, heat-stable, orally bioavailable small-molecule inhibitors of the dominant toxin classes, such as 3FTxs, PLA_2_s, and metalloproteases, could, in principle, be deployed at the community level to delay the progression of envenoming effects, an approach increasingly endorsed in the snakebite research community [3,77,80,83]. Overall, this study demonstrates the impact of spitting cobra venoms and their isolated 3FTxs on the haemostatic system and indicates the need for further detailed research into elapid venom-induced haemotoxic effects, an under-researched area in venom research. One limitation of the present study is that the findings are based solely on in vitro assays and therefore require validation in in vivo models and further investigation. Furthermore, although the study highlights a significant role for 3FTxs in platelet dysfunction, additional studies are needed to elucidate the precise molecular mechanisms by which these toxins exert their effects, in both the absence and presence of other venom toxins.

## 4. Methods

### 4.1. Venom Preparation

All snake venoms used in this study were purchased in lyophilised form from Latoxan (Valence, France) and stored at −80 °C. A stock solution of each venom (2 mg/mL) was prepared by dissolving it in phosphate-buffered saline (PBS) and stored at −20 °C until use. On the day of the experiment, the stock solution was diluted to prepare the required working concentrations of venom and used in appropriate experiments. *Crotalus atrox* venom was used as a positive control in selective assays.

### 4.2. Metalloprotease Assay

Metalloprotease activity in snake venoms was measured using a fluorogenic substrate, DQ-gelatin (ThermoFisher Scientific, Abingdon, UK), in accordance with the manufacturer’s instructions. DQ-gelatin was prepared in PBS as a 1 mg/mL stock solution. Several concentrations of cobra venoms (50–1.56 µg/mL) were added to a black 96-well microplate, followed by 2 µL (i.e., 2 µg/mL) of DQ-gelatin per well/reaction. The final reaction volume was adjusted to 100 µL with PBS. Fluorescence was measured at 10-min intervals for 90 min using a spectrofluorimeter (FLUOstar OPTIMA, Ortenberg, Germany) with excitation/emission wavelengths of 485/520 nm. For inhibition studies, marimastat (100–0.098 µM) and prinomastat (100–0.098 µM) (Sigma-Aldrich, Gillingham, UK) were preincubated for 10 min with the appropriate venom samples (50 µg/mL) prior to the addition of the substrate and the measurement of the resulting activity by spectrofluorimetry. PBS and *C. atrox* venom (50 µg/mL) served as the negative and positive controls, respectively.

### 4.3. Serine Protease Assay

Serine protease activity was measured using a fluorogenic substrate, Nα-benzoyl-L-arginine 7-amido-4-methylcoumarin hydrochloride (BAAMC; Sigma-Aldrich, UK), according to the manufacturer’s guidelines. BAAMC was prepared as a 2 mM stock solution in dimethyl sulfoxide (DMSO), and 2 µL (2 µM) was added to each well/reaction in a black 96-well plate after adding cobra venoms at concentrations ranging from 50 to 1.56 µg/mL. Fluorescence was recorded at multiple time points at excitation/emission wavelengths of 380/440 nm. Substrate in PBS and with *C. atrox* venom (50 μg/mL) served as the negative and positive controls, respectively.

### 4.4. Caseinolytic Assay

Caseinolytic activity was performed using azocasein (Sigma-Aldrich, UK). The azocasein solution was prepared by dissolving 5 mg in 1 mL of Tris-HCl buffer (50 mM, pH 8.0). Venom (10 µL, containing 20 µg) was mixed with 90 µL of substrate solution and incubated for 90 min at 37 °C. The reaction was terminated by adding 200 µL of trichloroacetic acid (TCA; 5% *v*/*v*), followed by centrifugation at 8000× *g* for 5 min. The supernatant (150 µL) was mixed with 0.5 M NaOH in a 96-well plate, and absorbance was measured at 440 nm using a spectrofluorometer. For the blank, Tris-HCl was used without venom.

### 4.5. PLA2 Assay

PLA_2_ activity in cobra venoms (50–1.56 µg/mL) was assessed using a chromogenic substrate, 4-nitro-3-(octanoyloxy)benzoic acid (NOB; Abcam, Cambridge, UK). NOB was prepared at a 3 mg/mL concentration in acetonitrile and then diluted in 10 mM Tris-HCl buffer (pH 8) to achieve a final substrate concentration of 0.25 mg/mL in assay wells. Reactions were carried out in 96-well microplates, and absorbance was measured at 440 nm using a FLUOstar OPTIMA spectrofluorometer (BMG Labtech, Germany). *Daboia russelii* venom (50 µg/mL; Kentucky Reptile Zoo, Bowen, KY, USA) and substrate alone served as positive and negative controls, respectively. For inhibition studies, venoms (50 µg/mL) were pre-incubated with varespladib (50–1.56 µM; Sigma-Aldrich, UK) for 5 min before substrate addition. All experimental conditions were performed in triplicate.

### 4.6. Fibrinogenolytic Assay

Metalloprotease inhibitors (100 µM) were mixed with human fibrinogen (1 mg/mL; Sigma-Aldrich, UK) in a final reaction volume of 200 µL, and samples were incubated at 37 °C. Aliquots of 30 µL were collected at 0, 1, 3, 6, 9 and 24 h and immediately mixed with an equal volume of 2× reducing sample treatment buffer [RSTB; 40% (*w*/*v*) SDS, 10% (*v*/*v*) β-mercaptoethanol, 20% (*v*/*v*) glycerine, 10% stacking gel buffer, and a small amount of bromophenol blue] and stored at −20 °C until use. Before analysis, samples were heated at 90 °C for 10 min and analysed by sodium dodecyl sulfate–polyacrylamide gel electrophoresis (SDS–PAGE).

### 4.7. SDS-PAGE

The 12% SDS-PAGE gels were prepared as follows. The resolving gel was prepared by mixing 30% (*w*/*v*) acrylamide–bisacrylamide mix (14 mL), resolving gel buffer (3 M Tris-HCl, pH 8.8; 4.38 mL), 10% (*w*/*v*) SDS (350 µL), 1.5% (*w*/*v*) ammonium persulphate (1.75 mL), distilled water (14.525 mL), and tetramethylethylenediamine (TEMED; 34 µL). The stacking gel was prepared using 30% (*w*/*v*) acrylamide–bisacrylamide mix (1.35 mL), stacking gel buffer (0.5 M Tris-HCl, pH 6.8; 2.5 mL), 10% (*w*/*v*) SDS (100 µL), 1.5% (*w*/*v*) ammonium persulfate (500 µL), distilled water (5.75 mL), and TEMED (8 µL). To analyse the protein profiles of collected venom fractions from RP-HPLC, each lyophilised fraction was dissolved in 100 µL of PBS, and 20 µL of each sample was mixed with 20 µL of RSTB. Samples were heated at 90 °C for 10 min, vortexed, and loaded onto 12% SDS–PAGE gels alongside Precision Plus Dual Colour Protein Standards (Bio-Rad, Watford, UK). Electrophoresis was performed at 70 V for 2 h using a Mini-PROTEAN electrophoresis system (Bio-Rad, UK). Following separation, gels were stained for 2 h with Coomassie Brilliant Blue solution [0.1% (*w*/*v*) Coomassie Brilliant Blue in 40% methanol and 10% acetic acid] and subsequently destained for 2 h using a destaining solution [10% (*v*/*v*) methanol and 10% (*v*/*v*) acetic acid] with gentle rocking.

### 4.8. Human Blood Collection and Preparation of PRP, Plasma, and Isolated Platelets

Human blood samples were obtained from healthy volunteers under ethical approval granted by the University of Reading Research Ethics Committee (UREC 17/17), after written informed consent was obtained from all donors prior to blood collection. Blood was collected by venipuncture into vacutainer tubes containing 3.25% (*w*/*v*) sodium citrate as an anticoagulant. PRP was prepared by centrifuging whole blood at 100× *g* for 20 min at 20 °C. The upper PRP layer was carefully collected without disturbing the buffy coat and allowed to rest at 30 °C before use. Platelet-poor plasma was obtained by centrifuging whole blood at 5000× *g* for 10 min at 20 °C; the clear supernatant was collected and allowed to rest at 30 °C for 30 min before use. Isolated platelets were prepared from PRP by adding acid citrate dextrose (ACD; 3 mL) and prostaglandin I_2_ (PGI_2_; 10 µL of a 125 µg/mL stock solution in ethanol). Samples were gently inverted and centrifuged at 1400× *g* for 10 min at 20 °C. The resulting platelet pellet was resuspended in modified Tyrode’s–HEPES buffer (1 mL, containing 5 mM glucose) with ACD (150 µL). The suspension volume was adjusted to 25 mL using pre-warmed modified Tyrode’s–HEPES buffer containing an additional 150 µL of ACD. A second centrifugation step was performed after adding 3 mL of ACD and prostacyclin (10 ng/mL) at 1400× *g* for 10 min at 20 °C. The supernatant was discarded, and the final platelet pellet was resuspended in modified Tyrode’s–HEPES buffer to achieve a final concentration of 4 × 10^8^ platelets/mL.

### 4.9. Platelet Aggregation

Platelet aggregation was performed by optical aggregometry using an aggregometer (Chrono-Log, Havertown, PA, USA). PRP was incubated at 37 °C with cobra venoms at concentrations ranging from 12.5 to 1.56 µg/mL, and light transmission was recorded for 5 min. Then, 5 µM ADP was added as an agonist. The baseline was adjusted to zero, and aggregation was monitored for a further 5 min.

### 4.10. Flow Cytometry Assay

The levels of fibrinogen binding and P-selectin exposure were measured by flow cytometry as markers of platelet activation. PRP samples were incubated with cobra venoms or their purified toxins (50–1.56 µg/mL) in the presence of an antibody master mix (35 µL). The master mix comprised antibodies against P-selectin (PE-Cy™5 mouse anti-human CD62P, 2 µg/reaction) and fibrinogen (polyclonal rabbit anti-human fibrinogen/FITC, 2 µg/reaction), diluted in 31 µL of HEPES-buffered saline (2.9 mM KCl, 134 mM NaCl, 0.34 mM Na_2_HPO_4_·12H_2_O, 1 mM MgCl_2_, 12 mM NaHCO_3_, and 20 mM HEPES; pH 7.3). After a 5 min incubation, platelet activation was induced by adding ADP (5 µM), thrombin receptor-activating peptide-6 (TRAP-6; 10 µM), or U46619 (4 µM), followed by a further 20 min incubation at 37 °C. Samples were then fixed with 0.2% (*v*/*v*) formyl saline prior to analysis. Flow cytometry was performed on a BD Accuri C6 flow cytometer (BD Biosciences, Wokingham, UK), with 5000 events acquired per sample within a platelet-gated region. Median fluorescence intensity was determined, and data were normalised to agonist-only controls, which were set to 100%. PRP alone served as the resting control.

### 4.11. Venom Fractionation

Cobra venoms were fractionated by RP-HPLC on a Spectra-Physics P200 HPLC system fitted with a BDS Hypersil C18 column (250 × 4.6 mm) (Thermo Scientific, Loughborough, UK). Lyophilised venom (2 mg) was reconstituted in 200 µL of 0.1% (*v*/*v*) trifluoroacetic acid (TFA) in HPLC-grade water and clarified by centrifugation at 8000× *g* for 5 min. The resulting supernatant (200 µL) was manually injected onto the column. Chromatographic separation was performed at a flow rate of 1 mL/min over a total run time of 100 min using a stepwise gradient of buffer A (0.1% TFA [*v*/*v*] in water) and buffer B (0.1% TFA [*v*/*v*] in acetonitrile), with buffer B steadily increasing to 60% over approximately 70 min. Elution was monitored by UV absorbance at 215 nm, and fractions with absorbance greater than 0.05 were collected and stored at −20 °C for subsequent analyses.

### 4.12. Mass Spectrometry

Protein identification was performed by excising the relevant band from SDS–PAGE gels containing the purified protein from relevant fractions. Gel slices were sent to Alta Bioscience (Birmingham, UK) for in-gel tryptic digestion and mass spectrometric analysis. The resulting peptide mass data were analysed by comparison with entries in the UniProt protein database to determine the identity of the purified protein.

### 4.13. PT and aPTT Assays

Plasma coagulation parameters were assessed on a Ceveron T100 automated coagulation analyser (Technoclone, Vienna, Austria). Human plasma samples (40 µL) from multiple donors were incubated with spitting cobra venoms and their purified toxins at a final concentration of 1.56 µg/mL. PT and aPTT were determined using commercial reagents according to the manufacturer’s instructions. For PT measurements, tissue thromboplastin and calcium chloride (25 mM) were used, whereas aPTT measurements were performed using silica/sulfatide phospholipid reagents in the presence of 25 mM CaCl_2_.

### 4.14. Plasmin Assay

Plasmin activity was measured using a chromogenic plasminogen assay kit (Technoclone, Austria) on a Ceveron T100 fully automated coagulation analyser (Technoclone, Austria), in accordance with the manufacturer’s instructions. Citrated human plasma samples were incubated with cobra venoms at a final concentration of 50 µg/mL before analysis. Samples were diluted as specified in the assay protocol, then mixed with the streptokinase-containing reagent, and finally, the chromogenic substrate was added. Plasmin activity was determined kinetically at 37 °C by measuring absorbance at 405 nm.

### 4.15. Thrombin Assay

Thrombin activity was assessed using BAAMC as a fluorogenic substrate. Thrombin was added to assay wells at a final concentration of 5 U/mL. Selected venom fractions (50 µg/mL) were then added and incubated with thrombin for 30 min at 37 °C before substrate addition. The substrate was added to achieve a final concentration of 100 mM in each well. Thrombin activity was monitored on a microplate reader by recording fluorescence every 10 min at excitation/emission wavelengths of 380/440 nm. Thrombin and PBS with substrate served as positive and negative controls, respectively.

### 4.16. ROTEM Analysis

Thromboelastometry analysis was performed on a ROTEM Delta analyser (Werfen, Warrington, UK) to assess the effects of cobra venoms and their fractions on whole-blood clotting. Samples were tested at a final concentration of 1.56 µg/mL, and their effects on clotting initiated via the intrinsic and extrinsic pathways were measured using INTEM and EXTEM assays, respectively. For each assay, citrated human whole blood (300 µL) was incubated with the sample before the addition of pathway-specific reagents, according to the manufacturer’s instructions. Blood samples were recalcified using the Star-tem reagent (0.2 M CaCl_2_ in HEPES buffer, pH 7.4). Clotting was then triggered using intrinsic activators comprising partial thromboplastin phospholipids derived from rabbit brain and ellagic acid, or extrinsic activators containing recombinant tissue factor in combination with phospholipids and heparin.

### 4.17. Haemolytic Assay

Following PRP preparation, erythrocytes were collected from the lower, red blood cell-enriched fraction of centrifuged human whole blood. Erythrocytes were washed three times by resuspension in an equal volume of PBS followed by centrifugation at 2000× *g* for 2 min, with the supernatant discarded after each wash. Washed erythrocytes were then resuspended in PBS and incubated with cobra venoms (50 µg/mL) or selected purified proteins (50 µg/mL). Samples were incubated for specific time periods (0, 1, 6, and 24 h), after which they were centrifuged at 2000× *g* for 2 min. Aliquots (50 µL) of the resulting supernatants were transferred to a 96-well microplate, and haemolysis was quantified by measuring absorbance at 540 nm on a spectrofluorometer. PBS and 1% (*v*/*v*) Triton X-100 (Sigma-Aldrich, UK) served as negative and positive controls, respectively. To assess the effects of PLA_2_ and metalloprotease on venom-induced haemolysis, cobra venoms were incubated with varespladib (100 µM), prinomastat (100 µM), or a combination of both and the resulting activity was measured as detailed above.

### 4.18. LDH Cytotoxicity Assay

LDH release was assessed using a commercially available LDH cytotoxicity assay kit (Thermo Fisher Scientific, UK) according to the manufacturer’s instructions. For platelet cytotoxicity, isolated human platelets were incubated with cobra venom or selected purified proteins (50–6.25 µg/mL) in a 96-well plate for 45 min at 37 °C. After incubation, 25 µL of supernatant from each well was transferred to a fresh 96-well plate and mixed with an equal volume (25 µL) of LDH reaction mixture. Samples were incubated for 30 min at 37 °C in the dark, after which 25 µL of stop solution was added. Absorbance was measured at 490 nm with a reference wavelength of 680 nm using a spectrofluorometer (BMG Labtech, Germany). To evaluate cytotoxicity in AB1190 myoblast cells, cells were seeded into 96-well tissue culture plates (100 µL growth medium per well) and maintained at 37 °C in a humidified atmosphere containing 5% CO_2_. After replacement of the medium, cells were treated with cobra venom (50 µg/mL each) or the corresponding purified toxin (50 µg/mL) and incubated for 4 h. Supernatants (25 µL) were then transferred to a new 96-well plate, and LDH activity was quantified as described above. Triton X-100 [1% (*v*/*v*); Sigma-Aldrich, UK] was used as the positive control. PBS and cell culture medium served as negative controls for the platelet and AB1190 cell assays, respectively.

### 4.19. Cell Viability Assay

The effects of cobra venoms and their corresponding purified toxins on the viability of AB1190 myoblasts were assessed using an MTS cell proliferation assay (Promega, Southampton, UK). Cells were seeded at 5000 cells per well in 96-well plates containing 100 µL of growth medium and incubated overnight to allow adherence. The medium was then replaced with fresh growth medium, and cells were treated with either cobra venoms (50 µg/mL each) or the selected purified toxin (50 µg/mL each) for 24 h. After incubation, 10 µL of MTS reagent was added to each well, and plates were incubated for an additional 4 h at 37 °C in a humidified atmosphere containing 5% CO_2_. Absorbance was measured at 490 nm using a spectrofluorometer (BMG Labtech, Germany). All conditions were tested in triplicate. Fresh growth medium and Triton X-100 served as negative and positive controls, respectively. Cell viability was expressed as a percentage relative to the negative control.

### 4.20. Statistical Analysis

All statistical analyses were performed using GraphPad Prism (version 7.0; GraphPad Inc., San Diego, CA, USA). Ordinary one-way analysis of variance (ANOVA) was applied where appropriate, followed by Fisher’s least significant difference (LSD) post hoc test to assess statistical significance. Data are presented as mean ± standard deviation (SD). For experiments requiring multifactorial analysis, two-way ANOVA was performed using the same software. Post hoc comparisons were performed using Tukey’s test as necessary.

## Data Availability

The original contributions presented in this study are included in the article. Further inquiries can be directed to the corresponding authors.
